# Tick Diversity Associated with the European Brown Hare (*Lepus europaeus* Pallas, 1778) in Croatia: Geographic Patterns and Species Composition

**DOI:** 10.3390/ani16162533

**Published:** 2026-08-14

**Authors:** Krunoslav Pintur, Tomislav Dumić, Ema Gagović, Nera Fabijanić, Vedran Slijepčević, Josip Gulin, Marin Maras, Relja Beck

**Affiliations:** 1 Department of Wildlife Management and Nature Conservation, Karlovac University of Applied Sciences, Trg J. J. Strossmayera 9, 47000 Karlovac, Croatia; tomislav.dumic@vuka.hr (T.D.); vedran.slijepcevic@vuka.hr (V.S.); marin.maras@vuka.hr (M.M.); 2 Croatian Veterinary Institute, Savska cesta 143, PP 883, 10000 Zagreb, Croatia; gagovic@veinst.hr; 3 Croatian Hunting Association, Vladimira Nazora 63, 10000 Zagreb, Croatia; nera_az@yahoo.com; 4 National Park Krka, Trg Ivana Pavla II 5, 22000 Šibenik, Croatia; josip.gulin@npk.hr

**Keywords:** haplotypes, *Ixodes festai*, *Ixodes inopinatus*, Ixodes ricinus, *Ixodes ventalloi*, molecular identification, *Rhipicephalus bursa*, tick community

## Abstract

Ticks are hematophagous ectoparasites of major veterinary and public health importance, acting as vectors of numerous pathogens affecting wildlife, domestic animals, and humans. Data on tick species infesting lagomorphs in Croatia remain scarce. This study investigated the species composition and genetic diversity of ticks collected from European brown hares (*Lepus europaeus*) in Croatia during 2024–2025 using morphological and molecular methods. A total of 158 hares from 18 localities were examined, and ticks were detected on 145 individuals, resulting in an infestation prevalence of 91.8%. Morphological identification and molecular analyses revealed nine tick species. The tick community was dominated by *Ixodes ricinus* (59.1%; *n* = 821), followed by *Ixodes festai* (26.8%; *n* = 372) and *Rhipicephalus bursa* (9.6%; *n* = 133), together accounting for more than 95% of all collected ticks. Molecular analyses confirmed species identification and revealed 10 haplotypes of *I. ricinus*, eight of *I. festai*, two of *I. ventalloi*, and one in *I. inopinatus*. The results demonstrate that European brown hares are important hosts for diverse tick species in Croatia and provide baseline data for future studies on tick ecology, tick-borne pathogens, and wildlife health management.

## Simple Summary

European brown hares are one of the most common wild mammals in Croatia and play an important role in natural and agricultural ecosystems. However, little is known about the ticks that feed on them. Ticks are small blood-feeding parasites that can spread diseases to wildlife, domestic animals and people. The aim of this study was to identify the tick species parasitizing European brown hares across Croatia and to examine how their composition differs between geographical regions. Over a two-year period, 158 hares from 18 locations were examined, and ticks were found on more than 90% of them. A total of 1388 ticks belonging to nine different species were collected. The most common species was *Ixodes ricinus*, which is widespread throughout Europe, while several other species were more common in coastal and island areas. The study also showed that tick populations differ between continental and Mediterranean parts of Croatia. These findings provide the first comprehensive overview of ticks on hares in Croatia and improve our understanding of how tick populations are distributed in different environments. This information can support wildlife management, help monitor diseases carried by ticks, and contribute to protecting animal and public health.

## 1. Introduction

Ticks are obligate hematophagous ectoparasites of major ecological and epidemiological importance because they transmit a wide range of pathogens affecting wildlife, domestic animals, and humans [1,2]. Owing to their broad host spectrum and complex multi-host life cycle, ticks exploit diverse vertebrate communities inhabiting different ecosystems, thereby playing a fundamental role in the maintenance and transmission of numerous zoonotic and veterinary pathogens. Tick-borne diseases currently represent the most important group of vector-borne diseases in Europe, and their incidence and geographical distribution have expanded considerably over recent decades due to climate change, habitat modification, increasing wildlife populations, and intensified movement of animals and humans [3,4].

The European brown hare (*Lepus europaeus* Pallas, 1778) is one of the most widespread lagomorph species in Europe and an ecologically and economically important game species [5,6]. Native to Europe and western Asia, it has also been successfully introduced into numerous regions outside its natural range [5]. In Croatia, the European brown hare is the only widely distributed native lagomorph species and occurs throughout continental and Mediterranean regions, including numerous Adriatic islands [7]. Besides its ecological importance as a medium-sized herbivore influencing vegetation dynamics and as prey for numerous mammalian and avian predators, the European brown hare has increasingly been recognized as a potential reservoir, maintenance host, or indicator species for several zoonotic and wildlife pathogens, highlighting its importance within the One Health concept [8,9,10].

Numerous studies have demonstrated that the European brown hare is involved in the ecology of several important zoonotic pathogens. It is closely associated with the epidemiology of *Francisella tularensis*, the causative agent of tularemia, and may represent an important source of human infection in endemic areas [10,11]. Furthermore, lagomorphs have been shown to harbour diverse tick species and contribute to the circulation of several tick-borne pathogens, including *Rickettsia* spp., Crimean-Congo haemorrhagic fever virus (CCHFV), hepatitis E virus (HEV), and other emerging microorganisms circulating in Mediterranean ecosystems [12,13,14,15,16,17]. These findings emphasize that the epidemiological significance of the European brown hare extends far beyond its traditional role as a game species.

The epidemiological importance of the European brown hare is closely linked to its interactions with ticks. Both immature and adult developmental stages of several ixodid species parasitize hares, enabling them to contribute to the maintenance of local tick populations while simultaneously supporting the circulation of tick-borne pathogens within natural ecosystems [13,14,18].

Beyond their importance as vectors of pathogens, ticks are integral components of terrestrial ecosystems. As obligate ectoparasites, they participate in long-term host–parasite interactions and contribute to ecological processes by linking wildlife, domestic animals and their associated microorganisms within complex ecological networks [3,13,14,19,20]. Host specificity varies considerably among tick species; while some parasitize a broad range of vertebrates, others are closely associated with particular host taxa and therefore have limited veterinary or public health significance [19,21].

Despite the ecological and epidemiological importance of the European brown hare, information on tick infestations in this host remains surprisingly scarce throughout Europe.

The overview of previous studies presented in Table 1 demonstrates considerable geographic variation in hare-associated tick communities across Europe, reflecting differences in climate, habitat characteristics, host distribution, and regional tick fauna.

In Croatia, 26 ixodid tick species have been recorded to date, yet information on ticks parasitizing European brown hares is almost entirely lacking [27,28,29,30,31,32,33]. Krčmar [29] examined three hares from eastern Croatia without detecting ticks, whereas Jurković [30] molecularly confirmed the presence of *Ixodes ventalloi* on hares from the island of Vir. No comprehensive data are available from either continental or Mediterranean regions of Croatia, despite the country’s remarkable ecological diversity and its location at the crossroads of Central Europe and the Mediterranean, where distinct tick faunas overlap.

Accurate identification of tick species is essential for understanding host associations, vector ecology, and the potential circulation of tick-borne pathogens [20,21]. Although morphological identification remains the cornerstone of tick taxonomy, several species exhibit considerable morphological similarity, particularly during immature developmental stages [21,34,35,36,37]. Therefore, integrating morphological identification with molecular confirmation provides a more robust and reliable approach for documenting tick biodiversity and improving our understanding of tick distribution.

Therefore, the aim of the present study was to determine the prevalence, infestation intensity, and species composition of ticks parasitizing European brown hares collected from different regions of Croatia using both morphological and molecular identification methods. By providing the first comprehensive overview of tick species associated with European brown hares across continental and Mediterranean Croatia, this study contributes to a better understanding of hare–tick interactions, regional tick ecology, and the epidemiological role of this important wildlife species in southeastern Europe.

## 2. Materials and Methods

### 2.1. Study Area and Sampling Localities

The examined European brown hares originated from localities distributed across two major geographical regions of Croatia: continental Croatia and the coastal/island region of the Adriatic Sea.

**Figure 1:**
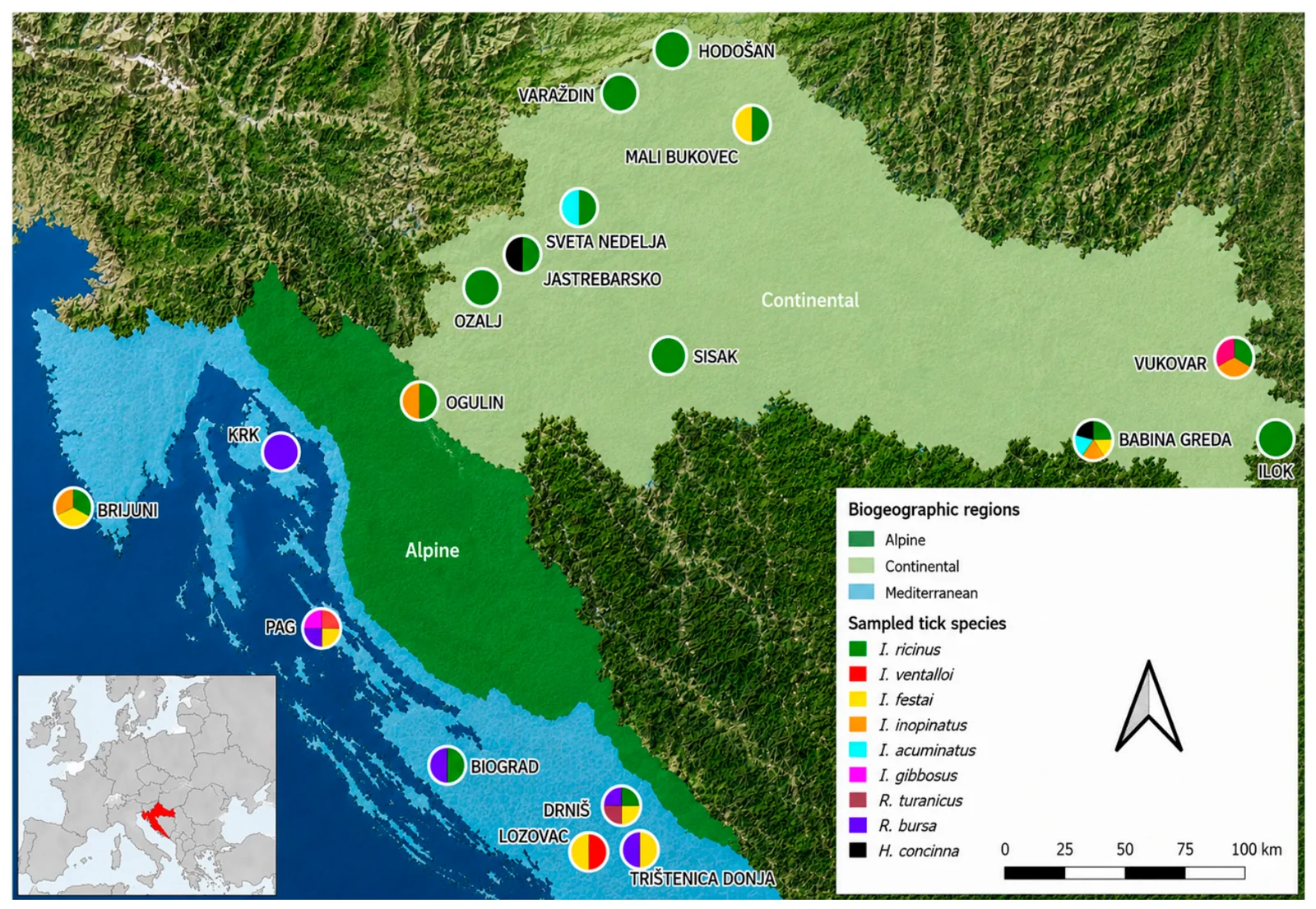
Geographic distribution of tick species collected across the biogeographic regions of Croatia.

A total of 158 European brown hares (including hunter-harvested and road-killed individuals) were collected from 18 localities across Croatia. Of these, 101 hares were collected in continental Croatia, whereas 57 originated from coastal and island localities (Figure 1). The number of examined hares varied among sampling localities.

The continental region included the localities of Babina Greda (*n* = 24), Hodošan (*n* = 9), Ilok (*n* = 6), Jastrebarsko (*n* = 22), Ogulin (*n* = 10), Ozalj (*n* = 1), Sisak (*n* = 1), Sveta Nedelja (*n* = 10), Varaždin (*n* = 8), Vukovar (*n* = 8), and Mali Bukovec (*n* = 2) (Figure 1). These areas comprise lowland agricultural landscapes, floodplain habitats, mixed deciduous forests, agricultural fields and fragmented grassland ecosystems typical of the Pannonian biogeographical region. Continental Croatia has a temperate continental climate and remains within the mid-latitude circulation belt throughout the year. Consequently, weather conditions are highly variable, with frequent and often pronounced atmospheric changes associated with diverse synoptic situations [38]. According to the Köppen climate classification, continental Croatia is predominantly characterized by humid temperate climate (Cfa and Cfb) [39].

The coastal and island region included the localities of the Brijuni Islands (*n* = 12), Krk Island (*n* = 1), Pag Island (*n* = 10), Drniš (*n* = 4), Lozovac (*n* = 17), Biograd (*n* = 3) and Trištenica Donja (*n* = 10) (Figure 1). These sites characterized Mediterranean and sub-Mediterranean climatic conditions, karst landscapes, rocky grasslands, shrub vegetation, maquis communities and fragmented agricultural habitats. The island localities are further distinguished by long-term geographic isolation and unique environmental conditions that may influence host–parasite interactions. The Adriatic coastal and island region is also influenced by mid-latitude atmospheric circulation during most of the year, resulting in frequent weather changes. During summer, however, the region comes under the influence of the Azores High, which limits the penetration of colder air masses and promotes stable weather conditions. The Adriatic Sea represents one of the most important climatic modifiers in this region, moderating temperature fluctuations and contributing to the development of a typical Mediterranean climate [39]. The coastal and island region is characterized by a Mediterranean climate with hot summers (Csa) [39].

### 2.2. Tick Sampling and Identification

Immediately after harvesting, each hare was individually placed in a transparent plastic bag and transported to the laboratory for examination. Road-killed hares were handled in the same manner immediately after being recovered. Individual plastic bags were used to minimize the loss of ticks that might detach from the host during transport. In the laboratory, each hare was systematically examined by visual inspection and palpation of the entire body surface, with particular attention to the head, ears, neck, axillary and inguinal regions, which are common attachment sites for ixodid ticks. Attached ticks were carefully removed using fine-tipped forceps, and any detached ticks found inside the plastic bags were also collected. All recovered ticks were preserved in 96% ethanol until morphological and molecular analyses.

Ticks were morphologically identified to species, sex, and developmental stage using standard taxonomic keys based on morphological characteristics [21,34,36,37].

Documentation was carried out using a SteREO Discovery. V20 microscope (Carl Zeiss AG, Oberkochen, Germany) equipped with AxioVision and ZEN 3.9 software. After identification, each tick was washed in 70% ethanol, rinsed with sterile distilled water, dried on sterile filter paper to remove external contaminants (such as soil particles, host-derived material, fur, and blood residues), and stored individually in sterile 2 mL tubes containing 96% ethanol until molecular analysis.

Following morphological identification, 293 representative specimens were selected for molecular confirmation using a stratified sampling approach. Representatives of each morphologically identified species were selected from all localities in which the species occurred and, whenever possible, from different developmental stages. The number of specimens analysed broadly reflected the abundance of each species in the overall dataset, while additional specimens were intentionally included for morphologically similar taxa, particularly *I. festai* and *I. ventalloi*, to verify species identification.

Genomic DNA was extracted using the MagMAX™ CORE Nucleic Acid Purification Kit (Thermo Fisher Scientific, Waltham, MA, USA) on the KingFisher™ Flex Purification System (Thermo Scientific), according to the manufacturer’s instructions, with a final elution volume of 100 μL. Species identity was confirmed by amplification and sequencing of a 460 bp fragment of the mitochondrial 16S rRNA gene using the forward primer 16SF (5′-CTGCTCAATGTTTTTTAAATTGCTGTGG-3′) and reverse primer 16SR (5′-CCGGTCTGAACTCAGATCAAGT-3′) [40].

PCR reactions were performed in a final volume of 20 µL containing 10 µL GoTaq^®^ G2 Master Mix (Promega, Madison, WI, USA), 7.2 µL DNase/RNase-free distilled water (Promega), 0.4 µL of each primer (10 pmol/µL), and 2 µL DNA template. Positive and negative extraction controls were included in all amplification rounds to monitor contamination.

Amplification products were evaluated by capillary electrophoresis using a QIAxcel system (Qiagen, Germany) with the QIAxcel DNA Fast Analysis kit (QIAGEN, Hilden, Germany; Cat. No. 929008), DNA QX Alignment Marker (15 bp–3 kb), and QX DNA Size Markers (50 bp–1.5 kb and 100 bp–2.5 kb). PCR products were purified using ExoSAP-IT™ PCR Product Clean-up Reagent (Applied Biosystems™, Thermo Fisher Scientific, Waltham, MA, USA; Cat. No. 78202) according to the manufacturer’s instructions and sequenced bidirectionally at Macrogen Europe (Amsterdam, The Netherlands) using the corresponding primer sets.

Raw sequence chromatograms were assembled and edited using SeqMan Pro version 18 and SeqBuilder Pro version 18 (DNASTAR, Madison, WI, USA). Consensus sequences were aligned and analysed using SeqMan and EditSeq version 18 (Lasergene, DNASTAR, Madison, WI, USA) and compared with reference sequences available in GenBank using the BLASTn algorithm (https://blast.ncbi.nlm.nih.gov; accessed on 20 June 2026) to confirm species identity.

### 2.3. Statistical Analysis

Statistical analyses were performed using the open-source statistical software R (accessed on 30 June 2026). Data were first checked, organized and summarized in Microsoft Excel, and all statistical analyses were subsequently performed in R version 4.5.1 (R Foundation for Statistical Computing, Vienna, Austria).

Parasitological descriptors followed the definitions of Bush et al. [41]. Tick prevalence was defined as the percentage of examined hares infested with one or more ticks, whereas mean intensity was calculated as the average number of ticks per infested hare.

Differences in infestation prevalence among localities and geographical regions were assessed using Pearson’s chi-square (χ^2^) test. When expected cell frequencies were low, Fisher’s exact test was applied. Differences in infestation intensity and tick abundance among localities and regions were evaluated using the non-parametric Kruskal–Wallis test, as tick count data were not normally distributed. The distribution of tick species was compared among sampling localities and geographical regions using χ^2^ tests. Developmental-stage composition was analysed overall and separately for each tick species. Associations between tick species and developmental stages were also assessed using χ^2^ tests.

Haplotype frequencies were calculated separately for each tick species. The distribution of haplotypes was analysed according to tick species, sampling locality and geographical region in order to evaluate potential geographical clustering and population structuring. Differences in haplotype distribution among regions and localities were tested using χ^2^ tests or Fisher’s exact test when appropriate.

The chi-square tests reported in this study were used as pre-specified overall (omnibus) tests of association and were therefore not adjusted for multiple comparisons. When post hoc analyses were performed to identify the categories contributing most strongly to significant overall tests, adjusted standardized residuals were examined with Holm correction. For significant Kruskal–Wallis tests, pairwise post hoc comparisons were performed using Dunn’s test with Holm correction.

## 3. Results

A total of 158 European hares were examined for tick infestation, of which 145 were infested, resulting in an overall infestation prevalence of 91.8%. In total, 1388 ticks were collected. The mean infestation intensity was 9.57 ticks per infested hare (median = 5). Mixed infestations with two or more tick species were recorded in 23 hares (14.6%).

Nine tick species were detected. *Ixodes ricinus* was the most prevalent species, with 821 specimens (59.1%), followed by *Ixodes festai* (372; 26.8%), *Rhipicephalus bursa* (133; 9.6%), *Ixodes ventalloi* (33; 2.4%) and *Ixodes inopinatus* (15; 1.1%). Less frequent findings included *Ixodes gibbosus* (7; 0.5%), *Rhipicephalus turanicus* (3; 0.2%), *Haemaphysalis concinna* (2; 0.1%) and *Ixodes acuminatus* (2; 0.1%).

Developmental-stage analysis showed that nymphs predominated, representing 718/1388 ticks (51.7%), followed by adults (639; 46.0%) and larvae (29; 2.1%). Among ticks with available sex data, females were more frequent (421; 66.6%) than males (211; 33.4%). Most ticks with available engorgement status were engorged (1064/1165; 91.3%), while non-engorged ticks represented 8.7%.

The developmental-stage distribution differed significantly among tick species (χ^2^ = 1118.10, df = 16, *p* < 0.001). In *I. ricinus*, nymphs were slightly more common than adults (436 vs. 374), while *I. festai* was represented mostly by adults (220 adults, 144 nymphs, 8 larvae). *R. bursa* was detected predominantly as nymphs (124/133), with only larvae recorded among the remaining specimens. *I. ventalloi* was mostly represented by adults (29/33), whereas *I. inopinatus* was mainly adult (13/15).

Infestation prevalence differed significantly among localities (χ^2^ = 60.93, df = 17, *p* < 0.001). Significant differences were also found in infestation intensity among localities (H = 56.32, *p* < 0.001). The highest mean infestation intensity was recorded on Brijuni (30.5 ticks/hare), followed by Ozalj (25.0; one examined hare), Krk (16.0; one examined hare), Ogulin (13.1) and Pag (12.5). The lowest values were recorded in Hodošan (1 tick/hare).

When grouped by region, infestation prevalence was high in both regions, with 89.1% in continental localities and 96.5% in coastal/island localities. This difference was not statistically significant (χ^2^ = 1.74, df = 1, *p* = 0.187). However, infestation intensity differed significantly between regions (H = 9.44, *p* = 0.002), being markedly higher in the coastal and island region (mean intensity = 12.96; median = 7) than in continental localities (mean intensity = 7.50; median = 4.5).

Post hoc analyses were broadly consistent with the descriptive results. The significant differences in prevalence among localities were mainly associated with lower-than-expected prevalence in Hodošan, while pairwise comparisons did not remain significant after Holm correction. For infestation intensity, Brijuni showed significantly higher values than selected localities, whereas high mean values in Ozalj and Krk were interpreted cautiously due to the very small sample size. In developmental-stage analyses, *R. bursa* was mainly associated with nymphs, while *I. festai*, *I. ventalloi* and *I. inopinatus* were mainly associated with adults.

Tick species composition differed significantly among localities (χ^2^ = 1815.27, df = 136, *p* < 0.001) and between continental and coastal/island regions (χ^2^ = 806.49, df = 8, *p* < 0.001). *I. ricinus* predominated in continental localities (649/675 ticks; 96.1%), whereas coastal/island localities showed a more diverse tick fauna, dominated by *I. festai* (367/713; 51.5%), followed by *I. ricinus* (172; 24.1%) and *R. bursa* (131; 18.4%). *I. ventalloi* was detected only in coastal/island localities.

Haplotype distribution also showed significant regional structuring (χ^2^ = 846.48, df = 18, *p* < 0.001). The most frequent haplotypes were *I. ricinus* H12 (53 specimens), *I. ricinus* H7 (35), *I. ricinus* H15 (21), *I. festai* H2 (20), *I. ricinus* H2 (18), *I. ricinus* H8 (16) and *I. festai* H1 (16). Several haplotypes showed marked geographical clustering. For example, *I. ricinus* haplotypes were dominant in continental localities such as Babina Greda, Jastrebarsko, Ogulin and Vukovar, whereas *I. festai* haplotypes were concentrated mainly in coastal/island localities, particularly Brijuni, Lozovac, Pag and Trištenica Donja. *I. ventalloi* haplotypes were detected only in coastal/island localities, especially Pag and Lozovac.

Seasonal analysis showed clear temporal variation in tick abundance and species composition. During spring, 13 hares were examined and all were infested, with 157 ticks collected, almost exclusively *I. ricinus* (156/157). During summer, infestation prevalence was also 100%, with 86 ticks collected, again dominated by *I. ricinus* (77/86), with occasional *I. inopinatus* and *H. concinna.* The highest number of ticks was collected during autumn–winter (1145 ticks from 139 hares), when species diversity was greatest. In this period, *I. ricinus* remained abundant (588 ticks), but *I. festai* (372), *R. bursa* (133), *I. ventalloi* (33), *I. gibbosus, I. inopinatus, I. acuminatus, H. concinna* and *R. turanicus* were also recorded.

Overall, the results demonstrate a very high level of tick infestation in European hares, with significant differences in infestation intensity, tick species composition and haplotype distribution among localities and regions. The data indicate that European hares may represent important hosts for immature and adult stages of several tick species, particularly *I. ricinus* in continental Croatia and *I. festai*, *R. bursa* and *I. ventalloi* in coastal/island regions.

Molecular analyses revealed considerable haplotype diversity among the collected ticks. The highest diversity was observed in *I. ricinus* and *I. festai*, whereas the remaining species were represented by a limited number of haplotypes or a single genetic variant.

A total of 10 haplotypes of *I. ricinus* (H1, H2, H7, H8, H10, H11, H12, H13, H15 and H16) were identified (Table 2). The most frequent haplotypes were H12 (*n* = 53), H7 (*n* = 35), H15 (*n* = 21), H2 (*n* = 18), H8 (*n* = 16), H10 (*n* = 11) and H11 (*n* = 10). Most *I. ricinus* haplotypes were recorded predominantly in continental localities, particularly Jastrebarsko, Ogulin, Babina Greda, Ilok, Sveta Nedelja and Vukovar. Haplotype H12 was the most widespread and prevalent haplotype, occurring in several geographically distant continental localities.

In contrast, *I. festai* showed a distinct geographical pattern. Eight haplotypes were identified (H1–H6, H10 and H11), with H2 (*n* = 20), H1 (*n* = 16), H5 (*n* = 13) and H10 (*n* = 11) being the most abundant (Table 2). Almost all *I. festai* haplotypes were restricted to coastal and island localities, particularly Brijuni, Lozovac, Pag and Trištenica Donja. The highest haplotype diversity of *I. festai* was recorded on Brijuni and in Lozovac, indicating possible long-term isolation and local diversification of tick populations in the Mediterranean region.

Two haplotypes were detected for *I. ventalloi* (H1 and H2), both restricted exclusively to coastal and island localities. Haplotype H2 (*n* = 5) was slightly more frequent than H1 (*n* = 3). Similarly, *I. inopinatus* was represented by a single haplotype, which was detected predominantly in continental localities.

The distribution of haplotypes differed significantly among regions (χ^2^ = 846.48, df = 18, *p* < 0.001), demonstrating strong geographical structuring of tick populations. Continental localities were characterized primarily by diverse *I. ricinus* haplotypes, whereas coastal and island localities were dominated by *I. festai*, *I. ventalloi* and *R. bursa*. This pattern suggests the existence of two distinct ecological assemblages associated with different climatic zones and host communities.

## 4. Discussion

To our knowledge, this study represents the first comprehensive survey of ticks parasitizing European brown hares in Croatia. By combining extensive geographical sampling with detailed morphological examination and molecular confirmation, our study substantially expands current knowledge of tick diversity in wild lagomorphs and demonstrates that Croatian hare populations harbour a tick assemblage markedly different from those reported elsewhere in Europe. These findings highlight Croatia as an important biogeographical transition zone where continental and Mediterranean tick faunas overlap.

The overall infestation prevalence of 91.8% demonstrates that European brown hares represent highly suitable hosts for ixodid ticks in Croatia. Such a high prevalence indicates frequent host–tick contact and suggests that hares contribute substantially to maintaining local tick populations. Comparable studies from Mediterranean Europe have also recognised lagomorphs as important hosts supporting both immature and adult stages of several ixodid species [13,14,18,24]. Nevertheless, the prevalence observed in the present study is among the highest reported for European brown hares, further emphasizing the ecological importance of this host within Croatian ecosystems. Together with previous evidence identifying European brown hares as reservoirs or sentinel hosts for *Francisella tularensis*, *Rickettsia* spp., Crimean–Congo haemorrhagic fever virus, hepatitis E virus and other emerging pathogens [9,10,11,12,13,14,15,16,17,18], our findings further reinforce the epidemiological importance of this species.

A total of nine tick species were identified, although the community was strongly dominated by *I. ricinus*, accounting for almost 60% of all collected specimens. As the most widespread tick species in Europe, *I. ricinus* exhibits remarkable ecological plasticity and parasitizes a broad spectrum of vertebrate hosts [42]. Numerous studies have recognised European brown hares as important hosts contributing to the maintenance of local *I. Ricinus* populations, particularly in ecosystems where alternative large mammalian hosts are less abundant. This observation is consistent with findings from Türkiye, where ticks constitute the predominant ectoparasites of European brown hares, further supporting the role of hares in maintaining ixodid populations across diverse ecological settings [22].

However, the Croatian tick fauna associated with European brown hares differs considerably from those described elsewhere in Europe. On the Iberian Peninsula, hare-associated tick communities are dominated by *R. pusillus*, *R. sanguineus* s.l., *H. lusitanicum*, *H. hispanica* and *I. ventalloi* [13,14,18]. In Italy, studies on the Italian hare (*Lepus corsicanus*) identified predominantly *I. ricinus*, *R. turanicus* and *Hyalomma* spp. [16,24], whereas *H. anatolicum* predominated on European brown hares in Greece [23]. In northern Europe, *I. ricinus* and *I. persulcatus* are the principal species associated with hares [43,44]. In contrast, Croatian hares harboured a unique assemblage comprising *I. ricinus*, *I. festai*, *I. ventalloi*, *I. inopinatus*, *R. bursa*, *R. turanicus*, *I. gibbosus*, *H. concinna* and *I. acuminatus*. To our knowledge, such a species composition has not previously been documented in European brown hares and most likely reflects Croatia’s unique position between Central European and Mediterranean biogeographical regions.

One of the most important findings of the present study is the pronounced geographical structuring of tick communities. Although infestation prevalence remained similarly high throughout the country, coastal and island localities exhibited significantly higher infestation intensity and considerably greater species diversity than continental Croatia. Whereas inland populations were almost exclusively dominated by *I. ricinus*, Mediterranean localities were characterised by the predominance of *I. festai*, together with frequent occurrences of *I. ventalloi* and *R. bursa*. Such clear regional segregation probably reflects differences in climate, vegetation, host communities and habitat characteristics and agrees with the well-established influence of Mediterranean climatic conditions on tick distribution [4]. The Adriatic islands may additionally represent isolated ecological systems where long-term host–parasite interactions have facilitated the persistence of distinct tick assemblages. Consequently, European brown hares appear to reflect regional differences in tick fauna remarkably well and may represent valuable sentinel hosts for monitoring changes in tick distribution associated with environmental and climatic changes.

Among all recorded species, the frequent occurrence of *I. festai* represents one of the most remarkable findings of the present study. Although *I. festai* has traditionally been regarded as a bird-associated tick occurring predominantly on passerine birds, particularly members of the family Turdidae, our study provides unequivocal molecular confirmation of its occurrence on European brown hares [36,45,46,47]. This finding substantially expands current knowledge regarding the host associations of *I. festai* and suggests that lagomorphs may represent more important maintenance hosts than previously recognised in Mediterranean ecosystems.

The significance of this finding has become even greater following the recent taxonomic revision of Mediterranean *Ixodes* species by Hornok et al. [36]. That study demonstrated that *I. festai* and *I. ventalloi* have frequently been confused because of their close morphological similarity and suggested that numerous published records of *I. ventalloi*, particularly those collected from passerine birds in Italy and Central Europe, most probably represent misidentified *I. festai*. Likewise, although *I. ventalloi* is generally regarded as a parasite of lagomorphs, rodents, carnivores and ground-dwelling birds [34,43,48], historical confusion between these closely related species has complicated interpretation of their true host ranges. Consequently, studies relying exclusively on morphology should be interpreted with caution.

Against this background, the integration of detailed morphological examination with mitochondrial 16S rRNA sequencing represents one of the principal strengths of the present study. Molecular confirmation demonstrated complete agreement with morphological identification and objectively confirmed the presence of *I. festai*, *I. ventalloi* and *I. inopinatus* on Croatian European brown hares. To our knowledge, this represents one of the few molecularly validated datasets confirming these three Mediterranean *Ixodes* species from wild lagomorphs and provides a robust taxonomic framework for future ecological, faunistic and epidemiological studies.

Importantly, our findings indicate that the apparent rarity of *I. festai* on mammals may partly reflect historical identification difficulties rather than genuine host specificity. As molecular approaches become increasingly incorporated into tick taxonomy, both the geographical distribution and host spectrum of this species may require substantial re-evaluation. The present study therefore not only confirms the occurrence of *I. festai* on European brown hares but also provides objective molecular evidence supporting its regular association with lagomorphs in Mediterranean environments.

The haplotype analyses further strengthened evidence for geographical structuring of Croatian tick populations. Whereas *I. ricinus* exhibited several widespread haplotypes throughout continental Croatia, *I. festai* and *I. ventalloi* showed geographically restricted haplotypes confined almost exclusively to Mediterranean and island localities. Particularly noteworthy was the relatively high haplotype diversity observed within *I. festai*, suggesting long-term persistence and local diversification of Mediterranean populations. Together with the clear geographical segregation of species, these findings indicate that continental and Mediterranean Croatia harbour partially independent tick populations shaped by different climatic conditions, host communities and evolutionary histories.

The predominance of nymphs and adults among collected ticks probably reflects both seasonal sampling and the biology of the recorded species. More than 90% of collected specimens were fully engorged, demonstrating that European brown hares represent highly suitable hosts capable of supporting successful blood feeding of several tick species. This observation further supports the hypothesis that hares actively contribute to maintaining local tick populations rather than serving merely as incidental hosts. Given their relatively limited home range and strong habitat fidelity, European brown hares may have potential as sentinel hosts for monitoring regional tick fauna and changes in tick distribution associated with ongoing environmental and climatic changes.

Although the present study covered a broad geographical area over two consecutive years, several limitations should be acknowledged. Sampling intensity varied among regions, and the unequal sample sizes among sampling localities may have influenced the detection of geographical differences, particularly in localities represented by a small number of examined hares. Furthermore, pathogen screening was beyond the scope of the present investigation.

Nevertheless, by integrating extensive field sampling with both detailed morphological examination and molecular confirmation, the present study establishes a robust baseline dataset for future investigations of tick ecology in Croatia. Future studies integrating tick-borne pathogen detection, population genetics and ecological analyses will provide further insight into the epidemiological significance and evolutionary relationships of the distinct tick communities identified here.

Overall, the present study provides considerably more than the first checklist of ticks parasitizing European brown hares in Croatia. It demonstrates that Croatian hare populations harbour a unique assemblage of continental and Mediterranean tick species that differs substantially from those reported elsewhere in Europe. Most importantly, the molecular confirmation of *I. festai*, *I. ventalloi* and *I. inopinatus* provides objective evidence for the occurrence of these taxonomically challenging species on European brown hares and establishes one of the first molecularly validated reference datasets for Mediterranean *Ixodes* species associated with wild lagomorphs [34,35,36,49]. In light of the recent taxonomic revision of *I. festai* and *I. ventalloi* [36], our findings considerably strengthen current knowledge of the host associations and geographical distribution of these neglected species and demonstrate the importance of integrating molecular tools into tick taxonomy. The clear geographical structuring of both species composition and haplotype diversity further indicates that Croatia represents an important contact zone between Central European and Mediterranean tick faunas.

Beyond their faunistic significance, these findings contribute to a broader understanding of the ecology of European ticks. They suggest that European brown hares are not only important maintenance hosts for several ixodid species but also valuable models for investigating the biogeography of Mediterranean tick communities. As molecular approaches continue to resolve long-standing taxonomic uncertainties within the genus *Ixodes*, datasets combining detailed morphology with DNA-based identification, such as the present study, will become increasingly important for redefining host associations, refining species distributions and improving our understanding of the ecology of neglected tick species.

## 5. Conclusions

In conclusion, this first nationwide study integrating morphological and molecular identification of ticks collected from European brown hares in Croatia suggests that European brown hares may contribute to maintaining local tick populations. Significant regional differences in species composition and haplotype distribution reveal strong geographical structuring of tick populations and emphasize the importance of considering biogeographical regions when studying tick ecology. Moreover, molecular confirmation of all identified species and the detection of geographically structured haplotypes provide valuable insights into tick diversity and evolution in southeastern Europe.

## Figures and Tables

**Table 1:** Tick species reported from hares in different European countries.

Tick Species	Host Species	Country
Argasidae	*Lepus europaeus*	Türkiye [22]
*Dermacentor* spp.	*Lepus europaeus*	Türkiye [22]
*Haemaphysalis* spp.	*Lepus europaeus*	Türkiye [22]
*Haemaphysalis hispanica*	*Lepus granatensis*	Spain (Iberian Peninsula) [13,14]
*Haemaphysalis parva*	*Lepus europaeus*	Türkiye [22]
*Hyalomma anatolicum*	*Lepus europaeus*	Greece [23]
*Hyalomma lusitanicum*	*Lepus granatensis*	Spain (Iberian Peninsula) [13,14,18]
*Hyalomma* spp.	*Lepus corsicanus*	Italy [16,24]
Ixodidae	*Lepus europaeus*	Türkiye [22]
*Ixodes persulcatus*	*Lepus timidus*	Sweden [25]
*Ixodes ricinus*	*Lepus corsicanus*	Italy [24]
	*Lepus europaeus*	Germany [26], Sweden [25]
	*Lepus timidus*	Sweden [25]
*Ixodes ventalloi*	*Lepus granatensis*	Spain (Iberian Peninsula) [13,14]
*Rhipicephalus pusillus*	*Lepus granatensis*	Spain (Iberian Peninsula) [13,14,18]
*Rhipicephalus sanguineus* s.l.	*Lepus granatensis*	Spain (Iberian Peninsula) [13,14,18]
*Rhipicephalus* spp.	*Lepus europaeus*	Türkiye [22]
*Rhipicephalus turanicus*	*Lepus corsicanus*	Italy [24]

**Table 2:** Molecular identification of tick haplotypes based on mitochondrial 16S rRNA sequences, sequence identity, and GenBank accession numbers.

Tick Species (Haplotype)	Per. Ident. (%)	Acc. Number	Our Acc. Number
*I. ricinus* (H1)	100	GU074593	PZ600149
*I. ricinus* (H2)	99.77	MH645522	PZ600150
*I. ricinus* (H7)	100	OY973604	PZ600151
*I. ricinus* (H8)	100	GU074645	PZ600152
*I. ricinus* (H10)	100	KF197124	PZ600153
*I. ricinus* (H11)	99.77	PV113058	PZ600154
*I. ricinus* (H12)	100	GU074630	PZ600155
*I. ricinus* (H13)	99.27	MK671585	PZ600156
*I. ricinus* (H15)	100	MK671589	PZ600157
*I. ricinus* (H16)	99.52	GU074589	PZ600158
*I. festai* (H1)	99.09	PZ177815	PZ599990
*I. festai* (H2)	98.42	PZ177815	PZ599991
*I. festai* (H3)	99.54	PZ177815	PZ599992
*I. festai* (H4)	98.86	PZ177815	PZ599993
*I. festai* (H5)	99.09	PZ177815	PZ599994
*I. festai* (H6)	99.3	PZ177815	PZ599995
*I. festai* (H10)	99.29	PZ177815	PZ599996
*I. festai* (H11)	98.38	PZ177815	PZ599997
*I. inopinatus* (H2)	100	GU074598	PZ600021
*I. acuminatus*	98.76	PV113079	PZ597521
*I. ventalloi* (H1)	99.55	KY231931	PZ600180
*I. ventalloi* (H2)	98.85	MW173473	PZ600181
*I. gibbosus*	99.76	OQ981354	PZ600008
*R. turanicus*	100	OP352773	PZ600218
*R. bursa*	99.76	MT302761	PZ600217

## Data Availability

The nucleotide sequences generated in this study have been deposited in the GenBank database under the accession numbers provided in the manuscript. The datasets supporting the findings of this study are available from the corresponding author upon reasonable request.
